# Metformin induces mitochondria-mediated and endoplasmic reticulum stress-mediated apoptosis and inhibits angiogenesis-related gene expression in breast cancer cells via targeting *VEGF-A/VEGFR2/NRP1*

**DOI:** 10.3325/cmj.2025.66.115

**Published:** 2025-04

**Authors:** Ares Alizade, Gulsah Evyapan, Ibrahim Seyfettin Celik, Berna Ozdem

**Affiliations:** 1 Department of Medical Pharmacology, Faculty of Medicine, Van Yuzuncu Yil University, Van, Turkey; 2 Department of Medical Biology, Faculty of Medicine, Van Yuzuncu Yil University, Van, Turkey; 3 Kahramanmaraş Health Services Vocational School, Kahramanmaraş, Turkey; 4 Department of Medical Biology and Genetics, İnönü University, Malatya, Turkey

## Abstract

**Aim:**

To investigate the apoptotic and anti-angiogenic effects of metformin in human MCF7 breast cancer cells.

**Methods:**

The effect of metformin on cell viability was assessed by MTS and crystal violet assays, and its effect on cell migration was evaluated by the wound healing assay. The gene expression and protein levels of angiogenesis- and apoptosis-related genes were determined by real-time polymerase chain reaction, Western blot, and flow cytometry.

**Results:**

Metformin reduced the viability and migration of breast cancer cells compared with the control group. Furthermore, metformin (10 μM) increased the apoptosis-related gene and protein expression of caspase-3, *Bax*, *AIF, CHOP* and *GRP78* 48 hours after treatment compared with the control group. In contrast, it significantly decreased *Bcl-2* and *Wee1* gene and protein expression and suppressed angiogenesis-related genes *VEGFA, VEGFR2*, and *NRP1*.

**Conclusions:**

Our results suggest that metformin treatment activates apoptosis pathways and inactivates the angiogenesis pathway. Although this study was conducted *in vitro* and did not directly evaluate blood vessel formation, the observed downregulation of angiogenesis-related genes suggests potential anti-angiogenic activity of metformin at the gene expression level.

Breast cancer is the most frequently diagnosed cancer in women (11.6%) and the leading cause of death due to cancer (1). The number of breast cancer cases has risen steadily over the past two decades (2). The majority of cancer chemotherapeutics exert their effects by inducing apoptotic death or by blocking cell cycle progression. Apoptosis, a crucial physiological process, is considered a vital mechanism for the selective clearance of cells and the regulated orchestration of cell death (3). By avoiding apoptosis, tumor cells resist chemotherapeutics (4). Therefore, the induction of apoptosis in tumor cells serves as a predictor for the response to tumor treatment. Moreover, many molecules, such as C/EBP-homologous protein (CHOP) and glucose-regulated protein (*GRP78*) are involved in the apoptosis pathway induced by endoplasmic reticulum (ER) stress. Activation of the unfolded protein response and the microenvironment of the tumor play a critical role in the development of adaptive responses (5-7). The majority of cancer-related deaths in individuals with solid tumors result from metastases. Migrastatic strategies present a distinctive therapeutic approach aimed at preventing all manifestations of cancer cell migration and invasion (8). On the other hand, tumor angiogenesis pertains to the development of novel blood vessels within solid tumors, facilitating the provision of nutrients and oxygen. This process promotes the dissemination of tumor cells, which is an important step in tumor development, progression, and metastasis (9,10). One of the best known regulators of angiogenesis is vascular endothelial growth factor (*VEGF*) (5), whose co-receptor is neuropilin 1 (*NRP1*). Preclinical evidence indicates that inhibiting *NRP1* not only suppresses tumor growth by impeding angiogenesis but also directly hinders tumor cell proliferation in specific models (11). Many cancers are associated with angiogenesis and are sensitive to anti-angiogenic treatments (12).

Mortality from breast cancer is increased in women with diabetes mellitus (DM), making DM a risk factor for breast cancer (13). Moreover, meta-analyses have substantiated a heightened risk of various cancers, including liver, pancreatic, colorectal, and breast cancers, in patients with DM (13,14). Metformin, an antihyperglycemic agent employed for several decades, has emerged as a primary pharmacological intervention for managing type 2 DM (T2DM). When used for the treatment of T2DM, metformin has been shown to reduce both cancer incidence and mortality, including that of breast cancer, compared with placebo or other antidiabetic medicines (15-17). Nevertheless, the mechanisms through which metformin mitigates tumor formation, particularly in the context of angiogenesis and apoptosis pathways, require further elucidation.

Cancer drug resistance is responsible for the majority of cancer recurrences and is a leading cause of cancer death (18). Apoptosis and angiogenesis have a fundamental role in cancer resistance to chemotherapeutics and in cancer development (4).

Currently, drugs are used to reduce the risk of breast cancer in premenopausal women. However, while these drugs are generally relatively well tolerated, they may lead to an increased risk of cardiovascular, endometrial cancer, venous thrombosis, pulmonary embolism, and stroke (6). Additionally, despite the initial positive responses, a major obstacle to effective treatment is developing long-term resistance to targeted therapies. Therefore, the effects of drugs on these mechanisms need to be better characterized in order to develop new, effective and alternative therapeutic drugs.

Previous studies have mostly investigated combination treatment including metformin, while our research specifically focused on metformin as a single agent and revealed its potential mechanisms via ER stress and *VEGFA/VEGFR2/NRP1* pathways. The aim of this study was to investigate the apoptotic and anti-angiogenic potential of metformin by assessing its effect on angiogenesis-related gene expression *in vitro* in MCF7 cells by targeting VEGF-A/VEGFR2/NRP1.

## MATERIALS AND METHODS

### Cell culture

The MCF7 cell line was purchased from the American Type Culture Collection (ATCC, Rockville, MD, USA). MCF7 cells were cultured in 10% FBS (Sigma-Aldrich, St. Louis, MO, USA), Dulbecco's Minimal Essential Medium (Thermo Fisher Scientific, Waltham, MA, USA), 1% PMSF, and 1% L-glutamine (Gibco, Thermo Fisher Scientific) in T25 flasks. Cells were incubated at 37 °C and 5% CO_2_. Every two days, the cell culture medium was changed.

### MTS assay

The 3-(4,5-dimethylthiazol-2-yl)-5-(3-carboxymethoxyphenyl)-2-(4-sulphophenyl)-2H-tetrazolium (MTS) assay was used to determine the effect of metformin (Thermo Fisher Scientific), on the growth of tumor cells *in vitro* and proliferation. The cells were seeded at 5000 cells/well in a 96-well plate containing 100-μL culture medium. They were cultured for 24, 48, and 72 hours in the presence/absence of metformin at various doses (350, 650, 1000, 2000, 5000, 7000, and 10 000 μg/mL). Then, 15 μL of MTS-labeling reagent (mg/mL) was added to the microtiter plate and incubated in a humidified atmosphere for two hours. After incubation, sample absorbance was measured by a Synergy HTX plate reader (BioTek Instruments Inc., Winooski, VT, USA) at 590 nm.

### Crystal violet assay

Crystal violet staining was used to monitor the effects of metformin on the viability of MCF7 breast cancer cells. Briefly, the cells were seeded on 24-well plates with 100 000 cells per well, and incubated overnight. The next day, the cells were exposed to metformin (1000, 2000, 5000, 7000, and 10 000 μg/mL), after which they were incubated in a humidified environment at 37 °C with 5% CO_2_ for 48 hours. On the day of staining, cells were stained with 0.5% crystal violet stain (Sigma-Aldrich) for 30 min at room temperature. After washing, the crystal violet dye was redissolved in 70% (v/v) ethanol/water, and absorbance was measured on a plate reader (Biochrom Ltd, Cambourne, Cambridge, UK) at 570 nm.

### Wound-healing assay

The MCF7 cells were plated in a 24-well culture plate at densities to achieve 70%-80% confluence after 24 h of growth. Next, a wound/scratch was created by cutting a straight line through the cell layer. After scratching, the cells were gently washed as a monolayer to remove any detached cells and replenished with fresh medium. They were subsequently exposed to different concentrations of metformin (0, 1000, 2000, 5000, 7000, and 10 000 μg/mL). Cell migration was photographed by phase-contrast microscopy (Leica, Wetzlar, Germany) at 0, 4, 8, 24, and 48 h. The wound closure was quantified using ImageJ software (US National Institutes of Health, Bethesda, MD, USA) by calculating the percentage of wound closure at each time point.

### Early and late apoptosis analysis by annexin V-PI flow cytometry

The cells were seeded in 6-well plates (20 × 10^4^ cells) a day before metformin was applied. After 24 and 48 h, the medium was placed into 15-mL Falcon tubes. The cells were extracted using trypsin and then centrifuged at 1500 rpm for 5 min. The Annexin V-PI Kit (BD Biosciences, San Jose, CA, USA) was used. For each cell, 400 μL of binding solution with a concentration of 1X was produced and then cooled on ice. Furthermore, for each cell, a 100-μL incubation mixture including 10 μL of 10X binding buffer, 10 μL of propidium iodine, 10 μL of annexin V, and 79 μL of distilled water was prepared. The solution was cooled and stored in a light-restricted environment for 10 min. The annexin V reagent was then retrieved from the refrigerated environment. Subsequently, a volume of 100 μL was added to each tube. The sample was incubated for 15 minutes at room temperature. A volume of 400 μL of 1X binding solution was added and allowed to stand for 30-60 min at room temperature. The resulting mixture was then analyzed using flow cytometry, according to the manufacturer’s protocol.

### Caspase-3 apoptosis activity assessment by flow cytometry

The cells were seeded into 6-well plates (20 × 10^4^ cells/well), a day before metformin was applied. After 24 and 48 h, the medium was placed into 15-mL Falcon tubes. The cells were extracted using trypsin and then centrifuged at 1500 rpm for 5 min. Next, they were resuspended in BD Cytofix/Cytoperm solution at a concentration of 1 × 10^6^ cells/0.5mL (fluorescein isothiocyante [FITC] Active Caspase-3 Apoptosis Kit; BD Pharmingen Inc., Franklin Lakes, NJ, USA). After incubation on ice for 20 min, the cells were washed twice with BD perm/wash buffer (1X) at a volume of 0.5 mL of buffer per 1 × 10^6^ cells at room temperature. The cells were resuspended in BD Perm/Wash buffer (1X), which had been prepared by diluting the stock solution according to the manufacturer’s instructions, together with the appropriate antibody. The resuspended cells were then incubated (30 min) and washed with 1.0 mL BD perm/wash buffer (1X). For flow cytometry analysis, the cells were resuspended in 0.5 mL BD perm/wash buffer (1X).

### Real-time polymerase chain reaction (RT-PCR) analysis

RT-PCR was used to assess the effect of metformin on the expression levels of several genes associated with apoptosis and angiogenesis pathways: *Bcl*-2, *caspase-3, Bcl-2*-associated X protein (*Bax*), *Wee1,* apoptosis-inducing factor (*AIF*), *CHOP, GRP78, VEGF A, VEGF* receptor 2 (*VEGFR2*), and *NRP1* in breast cancer cells. For this purpose, MCF7 cells were incubated in the absence or presence of metformin (500 mM) for 24 h and harvested. Total RNA was extracted with TRIzol Reagent (Invitrogen, Waltham, MA, USA), dissolved in deionized water treated with diethyl pyrocarbonate, and quantified using a spectrophotometer. cDNA was prepared using a cDNA kit (Thermo Fisher Scientific). The primers for the available genes are shown in Table 1. The cycle threshold values for the target genes and b-actin were obtained graphically.

**Table 1:** Primer sequences used for real-time polymerase chain reaction

Genes	Sequence (5′ → 3′)
***Caspase-3***	F: GAAATTGTGGAATTGATGCGTGA R: CTACAACGATCCCCTCTGAAAAA
***BAX***	F: CCCGAGAGGTCTTTTTCCGAG R: CCAGCCCATGATGGTTCTGAT
***BCL 2***	F: GGTGGGGTCATGTGTGTGG R: CGGTTCAGGTACTCAGTCATCC
***Wee1***	F: AGGGAATTTGATGTGCGACAG R: CTTCAAGCTCATAATCACTGGCT
***AIF1***	F: AAGTCAGACGAGAGGGGGTTA R: GCCAACTCAACATTGGGCT
***CHOP***	F: GGAAACAGAGTGGTCATTCCC R: CTGCTTGAGCCGTTCATTCTC
***GRP78***	F: GAAAGAAGGTTACCCATGCAGT R: CAGGCCATAAGCAATAGCAGC
***VEGFA***	F: AGGGCAGAATCATCACGAAGT R: AGGGTCTCGATTGGATGGCA
***VEGFR2***	F: GTGATCGGAAATGACACTGGAG R: CATGTTGGTCACTAACAGAAGCA
***NRP1***	F: ATCATTCAGGGTGGGAAGCA R: TGATGAATCGCGTGGAGAGA
***B-ACTIN***	F: TCTTCCAGCCTTCCTTCCTG R: CACACAGAGTACTTGCGCTC

### Cell homogenization

The cells treated with metformin for 24 and 48 hours were placed in tubes. The tubes were centrifuged (2000 rpm/4 °C/10 min), and the liquid was discarded. They were washed by adding 5 mL PBS and centrifuged (2000 rpm/4 °C/10 min). Then, PBS was removed. The cells in the tubes were mixed with RIPA buffer (Santa Cruz Biotechnology; 3 mL), PMSF (30 μL), sodium vanadate (30 μL), and protease inhibitor (30 μL). Homogenates were obtained from the cells using an ultrasonic homogenizer. The homogenates were centrifuged at 18 000 rpm for 15 minutes, after which the supernatants were collected.

### Protein quantification assay

The BCA protein assay kit (Abcam, Cambridge, UK) was used for protein quantification of homogenized cells. Bovine serum albumin was used to prepare a standard (1, 2, 3, 5, 7, 8, and 10 μg/mL concentrations [1 μg/mL]). Subsequently, 5 μL of each sample was diluted to a final volume with distilled water (100 μL). Following this, a working reagent mixture (prepared by combining Reagent A and Reagent B) (200 μL) was added to both the standard and the sample, and incubated for 30 minutes at room temperature. Then the absorbance was recorded (spectrophotometer, 562 nm). Protein concentrations were quantified in μg/μL using the Prism standard curve (GraphPad Software Inc., San Diego, CA, USA). To standardize Western blot experiments, protein quantification was performed.

### Western blotting

The cells were plated in six-well plates (20 × 10^4^ cells) a day before metformin was applied. After 24 and 48 h, the medium was placed into 15-mL Falcon tubes. The cells were extracted using trypsin and then centrifuged at 1500 rpm for 5 min. A polyvinylidene difluoride (PVDF) membrane (Merck Millipore) was used to transfer gel-electrophoresed proteins. For blocking, membranes were incubated with 5% non-fat dry milk in TBS-T for 1 hour at room temperature. The membrane was blotted and washed three times using TBS-T solution for 10 min each. Primary and secondary antibodies were incubated at 4 °C in a solution of 2.5% skimmed milk. Primary antibodies were used at a dilution of 1:500, and secondary antibodies at a dilution of 1:1000. The membranes were observed using the 733-2035 Vision Works LS image analysis, Gel Documentation Software 1 (Analytik Jena, Upland, CA, USA). Densitometry was conducted with the ImageJ program (version 1.51).

### Statistical analysis

The normality of distribution of continuous variables was tested with the Shapiro Wilk test. Differences in crystal violet and MTS results and those in gene expression between the control and treatment groups were evaluated using a one-way ANOVA with multiple comparisons, followed by Dunnett's *post-hoc* test. Differences in the wound healing assay results were assessed with a one-way ANOVA with Bonferroni correction. Differences in protein levels were assessed with a two-way ANOVA with Bonferroni correction. For the differences in Annexin V/FITC assay results, two-way ANOVA followed by Dunnett's multiple comparison tests was used. Differences in caspase-3 activity were assessed with two-way ANOVA with multiple comparisons and Dunnett's *post-hoc* test. Statistical analyses were performed with GraphPad Prism 8.0 (GraphPad Software Inc, San Diego, CA, USA) and Windows Office Excel (Microsoft, Redmond, WA, USA). *P* < 0.05 was considered statistically significant.

## RESULTS

### The effect of metformin on the viability and cell proliferation of MCF7 cells

**Figure 1:**
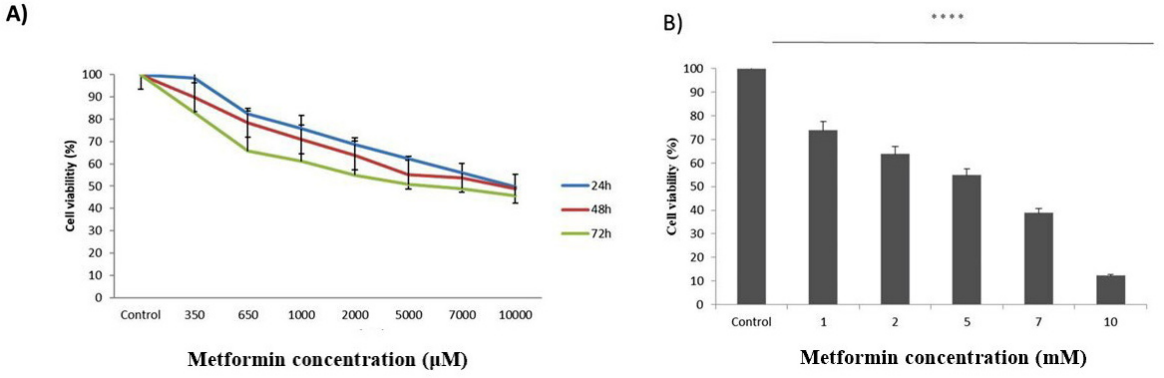
Effects of metformin on the growth and proliferation of breast cancer cells evaluated by (**A**) 3-(4,5-dimethylthiazol-2-yl)-5-(3-carboxymethoxyphenyl)-2-(4-sulphophenyl)-2H-tetrazolium (MTS) and (**B**) crystal violet assays. Data are expressed as the means of three separate experiments ± standard deviations (SD). ****P < 0.0001 compared with the control group (one-way ANOVA with multiple comparisons, followed by Dunnett's *post-hoc* test).

The cytotoxicity of metformin to MCF7 was evaluated using the MTS assay. The half-maximum inhibitory concentration (IC_50_) of metformin at 24 and 48 h was 7000 and 5000 μM, respectively. The cells exposed to various concentrations of metformin (350, 650, 1000, 2000, 5000, 7000, and 10 000 μM) for 24, 48, and 72 hours exhibited a significantly reduced viability within the 2000 to 10 000 μM range, in a time- and dose-dependent manner when compared with the control group (untreated cells) (Figure 1).

The metformin treatment remarkably suppressed the cell viability and cell proliferation as assessed by the crystal violet assay (*P* < 0.05) (Figure 1).

### The effect of metformin on inducing early and late apoptosis in MCF7 cells

**Figure 2:**
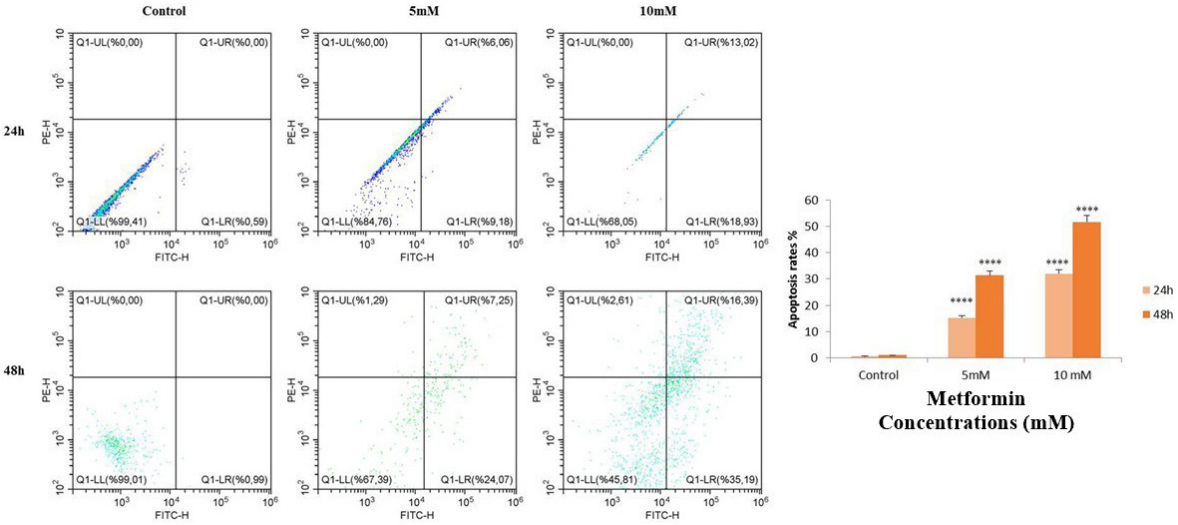
Early apoptosis, late apoptosis, and necrosis in MCF7 cells were determined at 24 and 48 h after treatment with metformin (5-10 mM) by flow cytometry. (Left) The MCF7 cells were stained with annexin V/FITC and propidium iodide (PI). UL: necrosis, LL: viability, UR: late apoptosis, LR: early apoptosis. (Right) The data are presented as means ±  standard deviations (SD), ****P < 0.0001. Each experiment was performed in triplicate (two-way ANOVA followed by Dunnett's multiple comparison tests).

The administration of metformin resulted in the initiation of both early and late stages of apoptosis, as assessed by Annexin V-PI staining. Early apoptosis is characterized by the presence of cells positive for annexin V and negative for PI, while late apoptosis is characterized by the presence of cells positive for both annexin V and PI. The overall apoptotic rate in cells subjected to metformin treatment at concentrations of 5 and 10 mM was 15.23% and 31.95%, respectively, at 24 h (Figure 2). At 48 h, the rates were 31.32% and 51.58%, respectively. The rate of apoptosis in the metformin-treated groups was significantly higher than that in the control group (*P* < 0.0001).

### The effect of metformin on inducing apoptosis through the upregulation of caspase-3 activity

Increased caspase-3 activity indicates the initiation of the apoptotic pathway in response to metformin administration. Breast cancer cells undergoing apoptosis while treated with metformin for 24 and 48 h were identified by the presence of caspase-3 activity using flow cytometry analysis.

**Figure 3:**
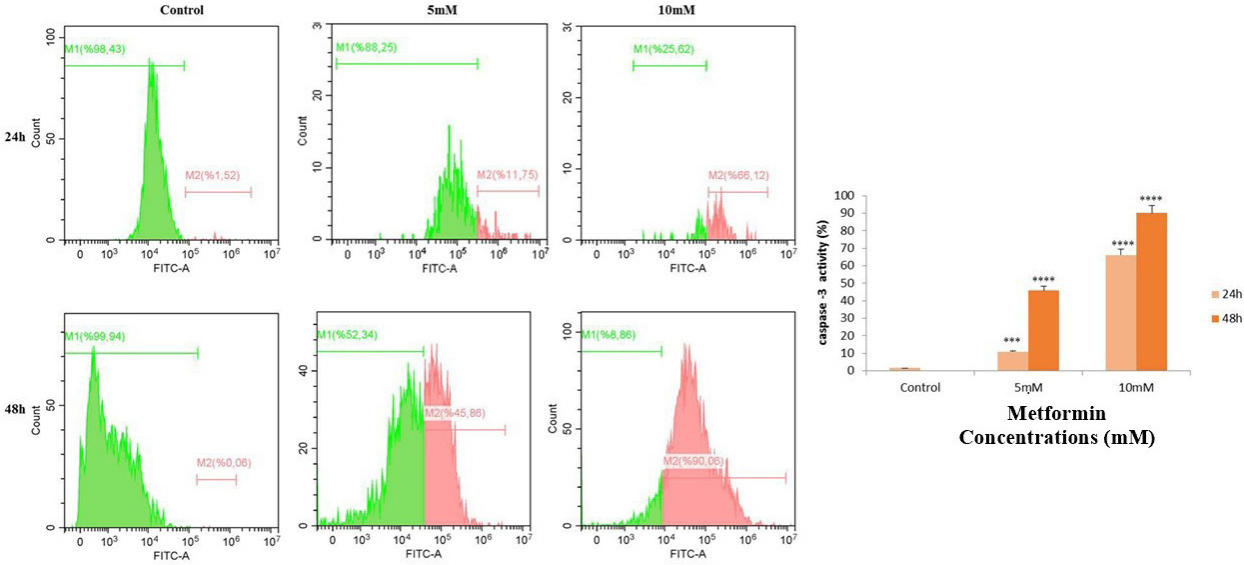
(Left) Caspase 3 activity was detected by flow cytometry after metformin treatment (5 and 10 μM) for 24 and 48 h. (Right) Each value represents the mean ±  standard deviation (SD) of three independent experiments, ***P < 0.001, ****P < 0.0001 (M1; caspase-3-negative cells, M2; caspase-3-positive cells) (two-way ANOVA with multiple comparisons and Dunnett's *post-hoc* test).

The administration of 5 mM and 10 mM metformin for 24 h resulted in a total caspase activity of 10.97% and 66.12%, respectively. After 48 h, the total caspase activity was 45.86% and 90.06%, respectively (Figure 3). Metformin administration significantly increased *caspase-3* activity compared with the control group (*P* < 0.001 and *P* < 0.0001, respectively).

### Metformin induced apoptosis through the downregulation of Bcl-2 and upregulation of Bax expressions

**Figure 4:**
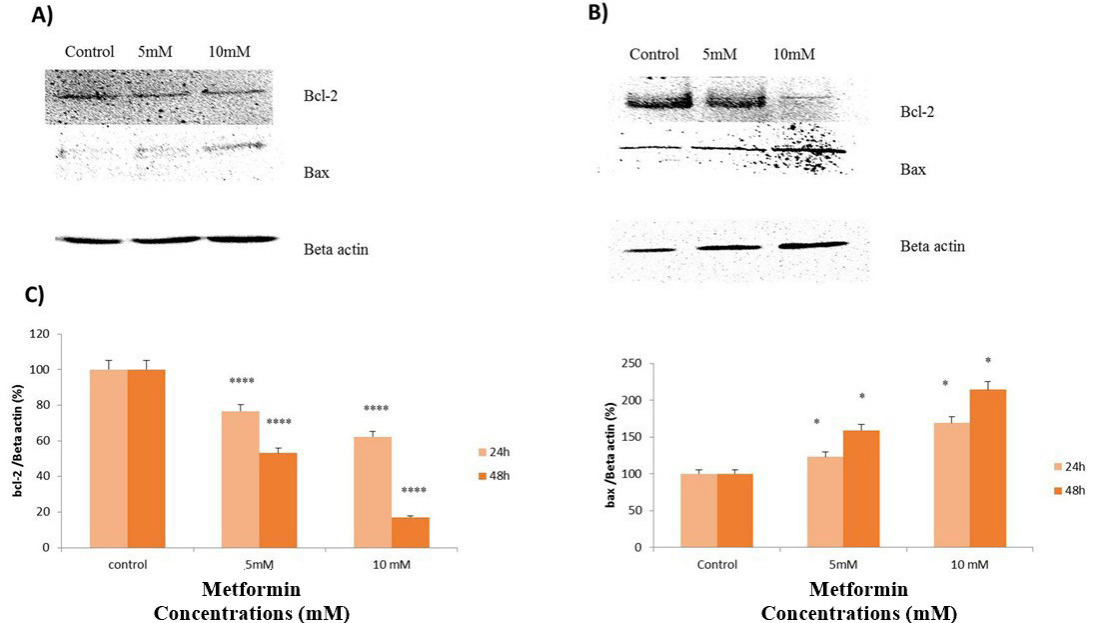
Western blot analysis of the apoptosis-associated proteins Bcl-2 and Bax in MCF7 cells treated with metformin. (**A**) *Bcl-2, Bax,* and *β-actin* protein bands at 24 and (**B**) 48 h. (**C**) Protein levels are expressed as a percentage of the *Bcl-2* and *Bax* levels in MCF7 cells. The data are expressed as the mean ± standard deviation, *P < 0.05, ****P < 0.0001 (two-way ANOVA with Bonferroni).

The expression levels of apoptotic markers in cells treated with metformin (5-10 Mm) for 24 and 48 h were also evaluated by Western blot analysis. *Bcl-2* and *Bax* proteins are associated with the process of apoptosis, and a change in *Bcl-2* and *Bax* protein levels indicates that apoptosis has occurred. Metformin decreased *Bcl-2* expression, increased *Bax* expression, and triggered apoptosis (Figure 4).

### The effect of metformin on inducing multiple apoptosis pathways

**Figure 5:**
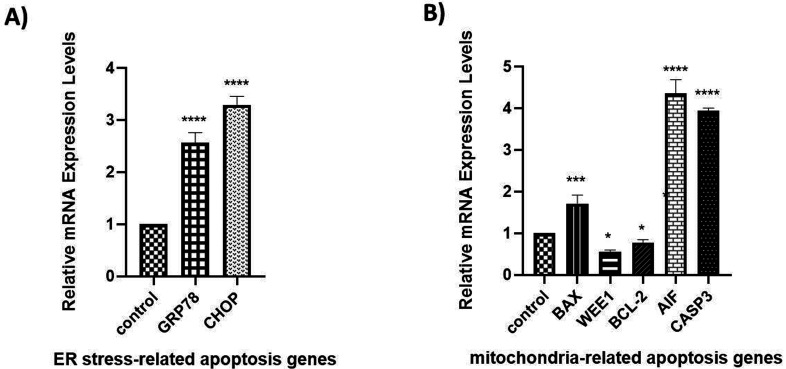
The effect of metformin (500 mM) on (**A**) endoplasmatic reticulum (ER) stress-related and (**B**) mitochondria-related apoptosis genes was detected by real-time polymerase chain reaction in MCF7 cells. The error bars represent the standard deviations in three experiments. *P < 0.05, ***P < 0.001, ****P < 0.0001 (one-way ANOVA, followed by Dunnett’s multiple comparison test).

Additionally, to investigate whether treatment with metformin induced apoptosis, the expression of genes associated with apoptosis was assessed by RT-PCR. Metformin significantly decreased the *Bcl-2* and *Wee1* gene expressions (Figure 5) compared with the control group, while it significantly increased the *caspase-3, AIF, CHOP*, and *GRP78* gene expressions.

### Metformin inhibits the migration of MCF7 cells

**Figure 6:**
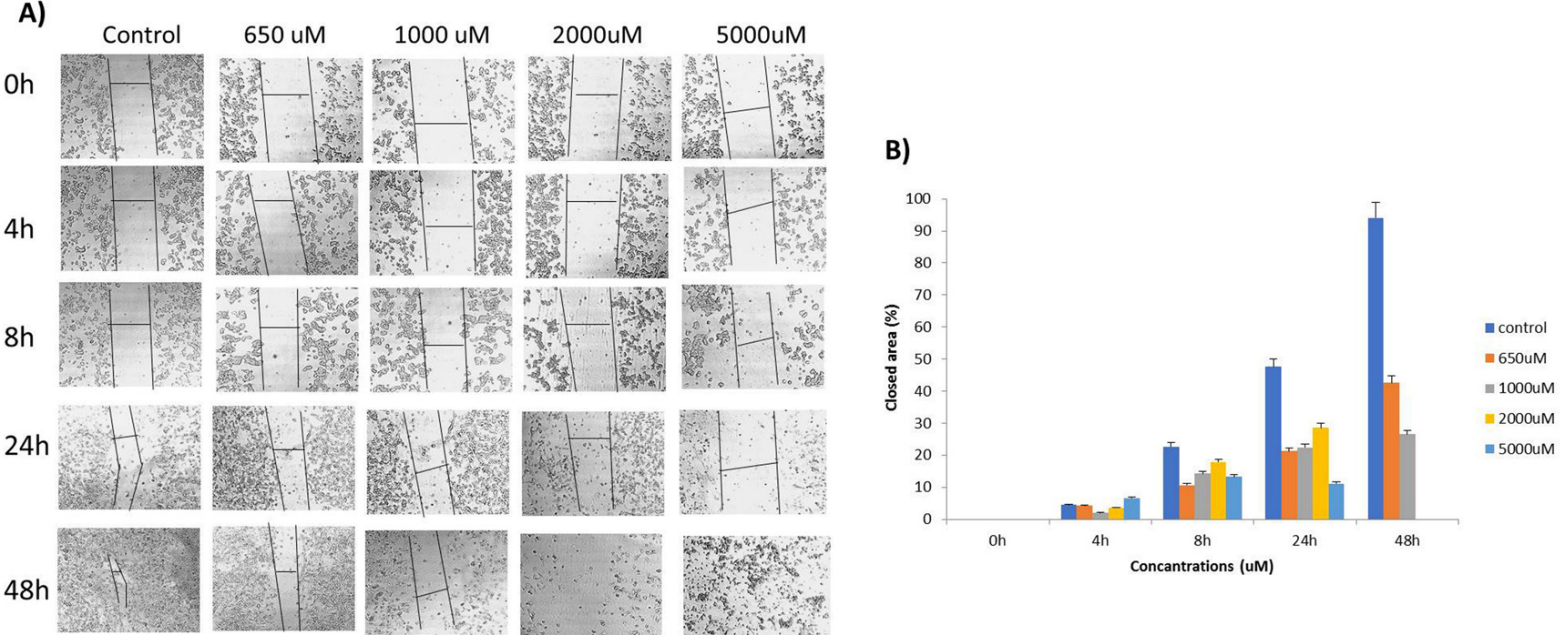
Metformin inhibited the MCF7 cell migration, shown by the wound healing assay. (**A**) Wound area comparison at 0, 4, 8, 24, and 48 h. (**B**) The wound closure rate (%) of cells in different treatment groups. The results are presented as the mean ± standard deviations of three independent experiments. ****P < 0.0001 (one-way ANOVA with Bonferroni correction).

The migration of breast cancer cells was evaluated using a wound healing assay. The migration of the treated cells to the entire wound area was substantially decreased compared with the control group (Figure 6).

### Metformin inhibits angiogenesis

**Figure 7:**
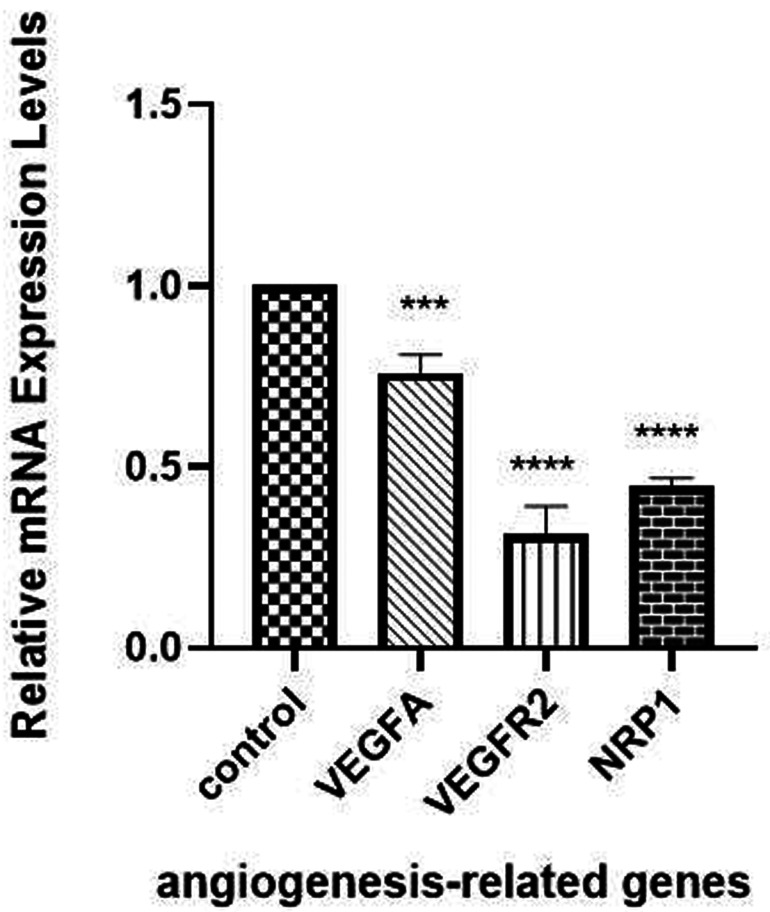
Metformin (500 mM) downregulated the gene expression of angiogenesis-related genes when compared with the control group. The mRNA expressions of *VEGFA, VEGFR2,* and *NRP1* were detected by real-time polymerase chain reaction. The results are presented as the mean ± standard deviations of three independent experiments. ***P < 0.001, ****P < 0.0001 (one-way ANOVA, followed by Dunnett’s multiple comparison test).

mRNA changes in the angiogenesis-related genes as a result of metformin application were assessed by RT-PCR. Metformin significantly reduced *VEGFA* (*P* < 0.001), *VEGFR2* (*P* < 0.0001), *and NRP1* (*P* < 0.0001) gene expression compared with the control group (Figure 7).

## DISCUSSION

Our *in vitro* experiments demonstrated that metformin significantly reduced cell viability and proliferation, inhibited migration capacity, and induced mitochondria- and ER stress-mediated apoptosis in MCF7 breast cancer cells. Furthermore, metformin treatment suppressed angiogenesis-related gene expression by downregulating *VEGFA*, *VEGFR2*, and *NRP1*, which highlights its potential to target both apoptotic and angiogenic pathways in breast cancer. Due to the developments in molecular and cellular biology, researchers have begun to elucidate the roles of growth factors, adhesion factors, and chemokines in tumor angiogenesis. Targeted therapeutic research based on these molecules has made anti-angiogenic therapy a promising strategy in anti-tumor treatment. Targeting apoptosis is one of the most successful methods of cancer treatment (14-16). Several molecular mechanisms have been proposed to explain metformin's apoptotic effects in breast cancer models. Dadashi et al demonstrated that a combination of metformin and digoxin-loaded nanoparticles exerted synergistic anticancer effects in MCF-7 breast cancer cells through the downregulation of critical oncogenic pathways, notably NOTCH-1 and HIF-1α gene expression (19).

Our study showed that metformin induced mitochondrial-mediated and ER stress-mediated apoptosis of breast cancer cells. Additionally, migration changes were investigated by wound healing assay. The inhibition rates of migration of MCF 7 cells were significantly time- and dose-dependent after 4, 8, 24, and 48 h of metformin treatment. Consistent with our findings, Yavuz et al reported that metformin, in combination with caffeic acid, significantly reduced cell migration and colony formation in MCF7 breast cancer cells (20).

Recent studies have shown that metformin significantly inhibited tumor angiogenesis. Furthermore, Hirsch et al reported that metformin suppressed inflammatory responses and factors promoting tumor angiogenesis (21). Consistent with our findings, Fatehi et al demonstrated that metformin enhanced the anti-cancer properties of resveratrol in MCF7 cells by inducing apoptosis and autophagy. However, unlike the combination treatment in their study, our research specifically focused on metformin as a single agent and revealed its potential mechanisms via ER stress and *VEGFA/VEGFR2/NRP1* pathways (22). Our study extends these findings by demonstrating that metformin alone can effectively suppress *VEGFA*, *VEGFR2*, and *NRP1* gene expression, indicating its potential as a single-agent anti-angiogenic treatment. However, there is a need to further clarify its effect on angiogenesis-related gene expression *in vitro* in breast cancer cells.

Since tumor angiogenesis is a complex, multi-step process, further investigations are necessary to elucidate additional molecular mechanisms involved. Our findings provide insight into the importance of the *VEGF-A/VEGFR2/NRP1* signaling pathway inhibition to improve anti-angiogenic therapy and suggest it could be a promising approach to breast cancer treatment. Although this study was conducted *in vitro* and did not directly evaluate blood vessel formation, the observed downregulation of angiogenesis-related genes (*VEGFA, VEGFR2, and NRP1*) suggests potential anti-angiogenic activity of metformin at the gene expression level.
